# Digitally Enabled Discharge Quality After Neurosurgical Traumatic Brain Injury: A 10-Year Cohort from a Brazilian Public Tertiary Center

**DOI:** 10.3390/healthcare14010032

**Published:** 2025-12-23

**Authors:** Roberto Salvador Souza Guimarães, Victoria Ragognete Guimarães, Carlos Marcelo Barros, Maísa Ribeiro Pereira Lima Brigagão, Francisca Rego

**Affiliations:** 1 School of Medicine, Universidade José do Rosário Vellano (UNIFENAS), Alfenas 37132-440, Minas Gerais, Brazil; victoriaragognete@gmail.com; 2 Department of Medicine, Federal University of Alfenas (UNIFAL-MG), Rua Gabriel Monteiro da Silva, 700, Centro, Alfenas 37133-840, Minas Gerais, Brazil; carlos.barros@unifal-mg.edu.br (C.M.B.); maisa.brigagao@unifal-mg.edu.br (M.R.P.L.B.); 3 Faculty of Medicine, University of Porto, Alameda Prof. Hernâni Monteiro, 4200-319 Porto, Portugal; mfrego@med.up.pt

**Keywords:** traumatic brain injury, discharge, electronic health records, clinical decision support, palliative care, patient education, quality improvement, Brazil

## Abstract

**Highlights:**

**What are the main findings?**

- In a 10-year cohort of 559 neur
- surgical TBI discharges at a Brazilian public tertiary center, warning sign counseling was documented in 16.1% (95% CI 13.2–19.5) and palliative care referrals were 0%.
- EHR documentation exposed specific, digitally fixable gaps in the discharge process that can be measured as process quality indicators.

**What are the implications of the main findings?**

- EHR discharge order sets with mandatory fields, CDS prompts for palliative care screening, and QR-coded patient handouts can standardize counseling and trigger appropriate referrals.
- The low, precisely estimated baseline provides a pragmatic target for quality improvement and a monitorable metric for resource-constrained hospitals.

**Abstract:**

**Background/Objectives:** Safe discharge after neurosurgical traumatic brain injury (TBI) depends on documented counseling and appropriate referrals, yet real-world fidelity is uncertain in resource-constrained settings. We quantified discharge process quality and identified digitally actionable gaps. **Methods:** The sample for this study was a retrospective cohort of 559 consecutive neurosurgical TBI patients discharged from a Brazilian public tertiary center (2012–2022). Data were abstracted from electronic health records. The primary outcome was documentation of warning sign counseling at discharge. Proportions are reported with exact Clopper–Pearson 95% confidence intervals. **Results:** The median age was 66 years (IQR 47–79.5); 78.5% were male and most received care under the public health system. Subdural hematoma predominated; hematoma drainage was the most frequent procedure. Warning sign counseling was documented in 16.1% of cases (89/559; 95% CI 13.2–19.5), and no palliative care referrals were recorded. **Conclusions:** A low baseline for a safety-critical discharge element exposes an immediately actionable target. Embedding discharge order sets with mandatory counseling fields in the EHR, clinical decision support prompts for palliative care screening and follow-up, and QR-coded patient handouts represent a pragmatic path to improve discharge quality and end-of-life readiness in the digital era.

## 1. Introduction

Traumatic brain injury (TBI) remains a major global cause of death and disability, with tens of millions living with long-term sequelae and marked cross-regional disparities in post-acute access and quality [1,2,3]. The hospital-to-home transition is a safety-critical juncture: gaps in discharge counseling, follow-up planning, and escalation instructions are repeatedly associated with avoidable emergency revisits and readmissions—particularly among older adults and after neurosurgical interventions [4,5,6]. Authoritative guidance converges on a concise set of high-value discharge elements (clear return precaution instructions in verbal and written form, medication reconciliation, contact pathways, timely information transfer to primary care), yet process fidelity and documentation quality remain inconsistent in routine care [4,5,6,7,8,9].

Within Brazil’s Unified Health System (SUS), traumatic brain injury represents a substantial public health burden, with a high volume of trauma-related admissions and considerable mortality and hospital costs concentrated in public tertiary hospitals. These centers function as regional trauma and neurosurgical referral hubs, and their discharge processes strongly influence long-term outcomes, particularly among older adults and caregivers who must manage complex neurological recovery at home. Understanding real-world discharge quality in such public tertiary settings is therefore critical to informing both national policy and local quality improvement efforts.

Digitally enabled workflows provide pragmatic levers to close these gaps. EHR-embedded discharge order sets with mandatory (hard-stop) fields improve the completeness and timeliness of summaries and standardize critical elements, reducing omission errors while preserving clinical flexibility [10,11,12,13]. Clinical decision support (CDS) can cue high-value actions “in the flow”—for instance, screening for palliative care needs in high-risk inpatients—using minimally intrusive prompts and sensible overrides [14,15]. Finally, patient-facing education delivered through validated content and QR-coded handouts strengthens comprehension and recall of danger signs and “what to do next”, complementing verbal counseling at the bedside and supporting safer self-management after discharge [6,8,9,16,17,18]. Evaluations of electronic discharge systems also show gains in usability and care coordination for older adults when content is structured and legible for downstream readers [19,20,21]. From a safety perspective, these interventions address known sentinel event failure modes concentrated in communication and handover processes [22].

Against this backdrop, we analyzed a 10-year neurosurgical TBI cohort at a Brazilian public tertiary center to quantify the fidelity of warning sign counseling documentation and the frequency of palliative care referral at discharge. We hypothesized that documentation rates would be low—defining a clear, digitally actionable baseline for quality improvement—and we report exact binomial confidence intervals appropriate for proportion estimates in this setting [23,24]. Our findings are interpreted in the context of pragmatic pathways to earlier, need-based palliative involvement in hospitalized, seriously ill patients [25,26,27], and within contemporary standards for adult mTBI discharge instructions and care transitions [5,28,29].

## 2. Materials and Methods

### 2.1. Study Design and Setting

We conducted a retrospective cohort study at a public tertiary hospital in Brazil with neurosurgical capabilities, evaluating consecutive neurosurgical TBI discharges from 2012 to 2022.

### 2.2. Participants

Eligible cases included adult patients (≥18 years) with traumatic brain injury (TBI) who required neurosurgical management (e.g., hematoma evacuation or decompressive craniotomy/craniectomy) and were discharged alive from the neurosurgical service during the study period (1 January 2012 to 31 December 2022). We excluded patients with non-traumatic neurosurgical conditions, patients younger than 18 years, patients who died during the index hospitalization, and cases in which the discharge summary was unavailable or had not yet been migrated to the electronic health record. After applying these criteria to all consecutive neurosurgical TBI admissions, 559 discharges met eligibility criteria and were included in the final cohort (*n* = 559).

### 2.3. Data Sources and Variables

We abstracted patient demographics (age, sex), payer/system status (public SUS vs. other), principal diagnosis (e.g., subdural hematoma, epidural/extradural hematoma, other TBI presentations), and index procedure (hematoma drainage; decompressive craniotomy/craniectomy). Discharge process variables included documentation of warning sign counseling (primary outcome) and palliative care referral (secondary process outcome).

### 2.4. Outcomes

The primary outcome was the documentation of warning sign counseling in the discharge summary. For abstraction purposes, warning sign counseling was operationally defined as explicit written documentation that the patient and/or caregiver had been informed about neurological danger signs after discharge (e.g., progressive or recurrent headache, worsening confusion, seizures, new focal weakness, repeated vomiting) and about when and where to seek urgent care if they occurred. Secondary outcomes were documentation of any specific warning sign content, documentation of return preparedness instructions (e.g., emergency contact routes, follow-up arrangements), and documentation of a palliative care referral at the time of discharge.

### 2.5. Statistical Analysis

Categorical variables are reported as counts and percentages. Continuous variables are summarized as medians with interquartile ranges and, secondarily, as means with standard deviations when informative. Exact two-sided Clopper–Pearson confidence intervals were used for binomial proportions [23,24]. Multivariable modeling was not prespecified or undertaken because key covariates—such as admission Glasgow Coma Scale, comorbidity burden, and discharge destination—were incompletely recorded in the electronic record for a substantial proportion of patients, and because the limited number of documented counseling events (*n* = 89) would have undermined the stability of adjusted models. Our analysis therefore focused on describing baseline process fidelity to inform future digital implementation efforts. Reporting follows established observational research reporting guidelines.

### 2.6. Software and Reproducibility

**Figure 1:**
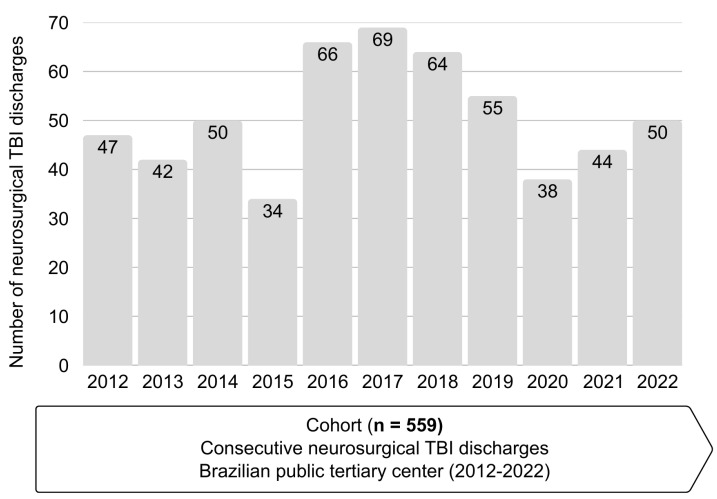
Yearly distribution of neurosurgical traumatic brain injury (TBI) discharges included in the cohort, by year of discharge (2012–2022; total *n =* 559).

Descriptive statistics and 95% exact binomial confidence intervals (Clopper–Pearson) were computed in R (v4.3.2; R Foundation for Statistical Computing, Vienna, Austria) using base functions from the stats package (e.g., binom.test); the results were cross-checked with the binom package (exact method). Figure 1 and the graphical abstract were prepared in Microsoft PowerPoint 365 (Microsoft Corporation, Redmond, WA, USA) and exported as high-resolution PNG files in accordance with MDPI figure requirements. No custom code was required; all analyses rely on standard, documented procedures available in the cited software.

### 2.7. Use of Generative AI

No generative AI systems were used to generate, analyze, or interpret data, or to create scientific content. A large language model was used exclusively for superficial language editing (grammar, spelling, punctuation, and formatting). All authors critically reviewed and approved the final text.

### 2.8. Ethics

This study was conducted in compliance with the Declaration of Helsinki and received approval from the UNIFENAS Research Ethics Committee (CAAE 74285923.2.0000.5143; approval 6.510.925). Due to the retrospective design and minimal risk involved, informed consent was waived.

## 3. Results

### 3.1. Cohort Characteristics

We included 559 consecutive neurosurgical TBI discharges spanning the 2012–2022 period. The median age was 66 years (IQR 47–79.5); 78.5% of patients were male, and 93.2% received care under the public health system (SUS). Subdural hematoma was the predominant diagnosis, and hematoma drainage was the most frequent surgical procedure.

### 3.2. Discharge Process Quality

Warning sign counseling was documented in 16.1% of cases (89/559; 95% CI 13.2–19.5). Crucially, no discharge record contained a documented palliative care referral (0.0%).

### 3.3. Table and Figure

Table 1 summarizes baseline characteristics and process measures in grouped blocks. Figure 1 displays the yearly distribution of neurosurgical TBI discharges included in the cohort (2012–2022; total *n* = 559).

## 4. Discussion

In this real-world neurosurgical TBI cohort (*n* = 559), warning sign counseling was documented in only 16.1% (95% CI 13.2–19.5), and no palliative care referrals were recorded at discharge. This pattern mirrors long-standing concerns regarding variability and omissions in discharge processes and after-care readiness across high-risk populations, particularly in serious illness and end-of-life trajectories [4,5,6,7,8,9]. Because documentation functions both as an aid to patient understanding and as an auditable artifact for measurement and feedback, low recorded fidelity represents more than a cosmetic shortfall: it constrains improvement cycles and blunts accountability for a safety-critical step [6,7,8,9,19,20,21,22].

Because our outcome measures are derived from electronic records, they capture recorded fidelity rather than directly observed bedside practice; some counseling or goals-of-care discussions may have occurred without being documented. As such, our estimates should be interpreted as a conservative, lower-bound approximation of true counseling rates. From a quality improvement perspective, however, documented counseling remains the actionable metric, because what is not recorded cannot be audited, fed back to clinicians, or reliably used to trigger downstream support.

From a bioethical standpoint, these findings raise concerns about how well discharge practice honors the principles of beneficence, non-maleficence, respect for autonomy, and justice. Beneficence and non-maleficence are threatened when high-risk patients and families leave the hospital without structured support to recognize neurological deterioration or to access urgent help, exposing them to potentially preventable harms in the home environment. Respect for autonomy is weakened when older adults with neurosurgical TBI are not offered systematic opportunities to revisit prognosis and goals of care, including the option of palliative care involvement along serious illness and potential end-of-life trajectories. Observational and interventional studies show that a substantial proportion of hospitalized patients meet criteria for palliative care assessment but are not referred, and that proactive, need-based screening and default palliative consultations improve symptom control, alignment between care and patient preferences, and family outcomes [25,26,27]. In resource-constrained settings, failing to identify and refer such patients also raises questions of distributive justice, as limited palliative care expertise is not preferentially directed to those with the greatest needs. Thus, the low rates of documented counseling and the absence of palliative care referrals in our cohort should be interpreted not only as quality deficits but also as signals that ethical obligations at the transition of care are not yet fully met.

For older adults with neurosurgical TBI and their caregivers, the lack of clearly communicated and documented warning signs and palliative care options can translate into uncertainty, fear, and a sense of abandonment as they navigate serious illness and potential end-of-life trajectories at home. Those with lower health literacy or weaker social networks are especially vulnerable to misunderstandings and delayed help-seeking, amplifying existing inequities. Future work should therefore also capture patient and family perspectives—through interviews or surveys about understanding of danger signs, confidence in managing at home, and perceived involvement in goals-of-care discussions—to complement the process metrics reported here.

Beyond their ethical salience, better-structured and better-documented discharge conversations have been associated in other high-risk populations with improved patient understanding, fewer discrepancies in post-discharge regimens, and reductions in preventable emergency visits or adverse events [5,8]. Although analogous evidence in neurosurgical TBI remains limited, the same mechanisms—clarified expectations, explicit safety-netting, and documented follow-up plans—are likely to operate in this cohort, particularly among older adults and their caregivers.

Our findings map directly onto digital process redesign opportunities at the intersection of discharge safety and serious illness care. First, EHR discharge order sets with mandatory fields can ensure that danger sign counseling is consistently captured and that the content of return precautions is standardized in line with guidance (e.g., progressive headache, confusion, seizures, new focal deficits, when/where to seek help) [10,11,12,13]. Work on human factors suggests that converting optional narrative fields into concise required items (with a documented reason for non-applicability) elevates reliability while minimizing free-text burden and re-work downstream [10,11,12,13,19].

Second, CDS prompts can automate palliative care screening for high-risk phenotypes (older age, severe injury/procedures, multimorbidity, recurrent admissions), increasing appropriate consults and earlier goals-of-care conversations without imposing excessive alert load when rules are judiciously designed [14,15,25,26,27]. Framing such prompts as need-based support—co-designed with neurosurgery and palliative teams—aligns with evidence for default-based strategies that overcome under-referral in serious illness [25,26,27].

Third, QR-coded patient handouts linking to validated, plain-language instructions (and multimedia where appropriate) provide a durable complement to verbal counseling, improving comprehension and retention and reducing version drift of educational materials; analytics from QR usage also enable light-touch monitoring of reach and engagement [6,8,9,16,17,18].

In Brazilian public tertiary hospitals, customization of EHRs may be constrained by heterogeneous or legacy systems, limited informatics capacity, and competing priorities for scarce IT resources. However, the interventions we propose are deliberately narrow in scope—adding a small number of mandatory counseling fields and simple rule-based prompts—rather than large-scale EHR redesign. Experience from general medicine and emergency services, including in public and resource-constrained settings, suggests that such co-designed, tightly focused CDS modifications are feasible and acceptable when aligned with clinicians’ workflows and supported by local leadership [30,31,32].

Key strengths include a consecutive, decade-long cohort from a public tertiary neurosurgical service; a priori process outcomes tied to patient safety and serious illness care; and the use of exact binomial intervals appropriate to the data [23,24]. Limitations reflect the retrospective design and documentation dependence: we measured recorded fidelity rather than directly observed bedside practice, and some counseling or goals-of-care discussions may have occurred without being documented. In addition, we did not assess downstream clinical outcomes (e.g., unplanned contacts, emergency returns, readmissions, mortality), which limits our ability to quantify the direct impact of the observed documentation deficits on patient safety; our findings should therefore be interpreted as defining a baseline process gap rather than establishing causal effects. Finally, several potentially important covariates—such as admission Glasgow Coma Scale, comorbidity burden, and discharge destination—were incompletely recorded in the electronic record, and the limited number of documented counseling events (*n* = 89) precluded stable multivariable modeling of predictors of counseling or referral. Prospective studies with richer covariate capture, linked outcome data, and mixed-methods approaches (including direct observations and clinician/patient interviews) are needed to further characterize bedside practice and determinants of discharge process quality in neurosurgical TBI [5,6,7,8,9,16,17,18,19,28,29].

## 5. Conclusions

Documentation of warning sign counseling at discharge was present in only 16.1% of neurosurgical TBI cases, revealing an immediate, actionable target for improvement. A simple digital redesign—EHR discharge order sets with required fields, in-flow CDS prompts for palliative care screening, and QR-coded patient instructions—can standardize counseling, trigger timely referrals, and strengthen after-care in resource-constrained settings, thereby operationalizing the bioethical principles of beneficence, non-maleficence, respect for autonomy, and justice at the point of discharge. These measures are low-cost, auditable within the EHR, and scalable across tertiary services. Future work should assess patient-centered outcomes (comprehension, unplanned returns, mortality) and implementation fidelity following deployment.

## Figures and Tables

**Table 1:** Baseline characteristics and discharge process measures (*n* = 559).

Characteristic		Value
Demographics	Age, years, median (IQR)	66.0 (47.0–79.5)
	Age, years, mean (SD)	61.0 (23.1)
	Male sex, %	78.5
	Care under SUS, %	93.2
Principal diagnosis	Subdural hematoma (SDH), %	61.2
	Epidural/extradural hematoma (EDH), %	14.9
	Other TBI presentations, %	10.0
Procedures	Hematoma drainage, %	82.2
	Decompressive craniotomy/craniectomy, %	18.9
Discharge process	Warning sign counseling documented, % (n/N; 95% CI)	16.1 (89/559; 13.2–19.5)
	Palliative care referral documented, % (n/N)	0.0 (0/559)

Abbreviations: TBI, traumatic brain injury; SUS, Brazilian Unified Health System; SD, standard deviation; IQR, interquartile range; CI, confidence interval; SDH, subdural hematoma; EDH, epidural (extradural) hematoma.

## Data Availability

The data presented in this study are available on reasonable request from the corresponding author. The data are not publicly available due to privacy and ethical restrictions imposed by the Research Ethics Committee of Universidade José do Rosário Vellano (UNIFENAS).

## References

[1] James S.L., Theadom A., Ellenbogen R.G., Bannick M.S., Montjoy-Venning W., Lucchesi L.R., Abbasi N., Abdulkader R., Abraha H.N., Adsuar J.C. (2019). Global, regional, and national burden of traumatic brain injury and spinal cord injury, 1990–2016. Lancet Neurol..

[2] Maas A.I.R., Menon D.K., Manley G.T., Abrams M., Åkerlund C., Andelic N., Aries M., Bashford T., Bell M.J., Bodien Y.G. (2022). Traumatic brain injury: Progress and challenges in prevention, clinical care, and research. Lancet Neurol..

[3] Dewan M.C., Rattani A., Gupta S., Baticulon R.E., Hung Y.-C., Punchak M., Agrawal A., Adeleye A.O., Shrime M.G., Rubiano A.M. (2018). Estimating the global incidence of traumatic brain injury. J. Neurosurg..

[4] Coleman E.A. (2003). Falling through the cracks: Challenges and opportunities for improving transitional care. J. Am. Geriatr. Soc..

[5] Agency for Healthcare Research and Quality (AHRQ) (2020). Strategy 4: Care Transitions from Hospital to Home (IDEAL Discharge Planning). https://www.ahrq.gov/patient-safety/patients-families/engagingfamilies/strategy4/index.html.

[6] National Institute for Health and Care Excellence (NICE) (2023). Head Injury: Assessment and Early Management (NG232).

[7] American College of Surgeons (ACS) (2023). Trauma Quality Programs Best Practices Guidelines: Geriatric Trauma/Transitions of Care.

[8] Jack B.W., Chetty V.K., Anthony D., Greenwald J.L., Sanchez G.M., Johnson A.E., Forsythe S.R., O’Donnell J.K., Paasche-Orlow M.K., Manasseh C. (2009). A reengineered hospital discharge program to decrease readmissions. Ann. Intern. Med..

[9] Kripalani S., LeFevre F., Phillips C.O., Williams M.V., Basaviah P., Baker D.W. (2007). Deficits in communication and information transfer between hospital-based and primary care physicians. JAMA.

[10] Mehta R.L., Pauly R.P., Chan C.T., Vercaigne L.M., Gauthier T.P., Perl J., Pierratos A., Silver S.A., Nesrallah G.E., Copland M. (2017). Assessing the impact of introducing an electronic discharge summary system. BMC Health Serv. Res..

[11] Schnipper J.L., Hamann C., Ndumele C.D., Liang C.L., Carty M.G., Karson A.S., Bhan I., Coley C.M., Poon E.G., Turchin A. (2009). Effect of an electronic medication reconciliation application and process redesign on potential adverse drug events: A cluster-randomized trial. Arch. Intern. Med..

[12] Davies G., Kean S., Chattopadhyay I. (2021). Improving the quality of electronic discharge summaries from medical wards: A quality improvement project. Future Healthc. J..

[13] Powers E.M., Shiffman R.N., Melnick E.R., Hickner A., Sharifi M. (2018). Efficacy and unintended consequences of hard-stop alerts in EHR systems: A systematic review. J. Am. Med. Inform. Assoc..

[14] Bright T.J., Wong A., Dhurjati R., Bristow E., Bastian L., Coeytaux R.R., Samsa G., Hasselblad V., Williams J.W., Musty M.D. (2012). Effect of clinical decision-support systems: A systematic review. Ann. Intern. Med..

[15] Bates D.W., Saria S., Ohno-Machado L., Shah A., Escobar G. (2014). Big data in health care: Using analytics to identify and manage high-risk and high-cost patients. Health Aff..

[16] Oh S., Choi H., Oh E.G., Lee J.Y. (2023). Effectiveness of discharge education using teach-back method on readmission among heart failure patients: A systematic review and meta-analysis. Patient Educ. Couns..

[17] Ma G., Jiang P., Miao C., Huang Y., Li H., Zhao Y. (2024). Association between pre-hospital e-education via QR code and hospital stay in inguinal hernia patients undergoing general anaesthesia: A retrospective study. J. Multidiscip. Healthc..

[18] Jesus T.S., Stern B.Z., Lee D., Zhang M., Struhar J., Heinemann A.W., Jordan N., Deutsch A. (2024). Systematic review of contemporary interventions for improving discharge support and transitions of care from the patient experience perspective. PLoS ONE.

[19] Callen J.L., Alderton M., McIntosh J. (2008). Evaluation of electronic discharge summary systems—A literature review. Int. J. Med. Inform..

[20] Tremoulet P.D., Shah P.D., Acosta A.A., Grant C.W., Kurtz J.T., Mounas P., Kirchhoff M., Wade E. (2021). Usability of Electronic Health Record–Generated Discharge Summaries: Heuristic Evaluation. J. Med. Internet Res..

[21] Cam H., Aydin A., Erdogan A. (2023). Communication at hospital discharge of older patients: A qualitative study. BMC Health Serv. Res..

[22] Patra K.P., Jeus O. (2023). Sentinel events. StatPearls.

[23] Clopper C.J., Pearson E.S. (1934). The use of confidence or fiducial limits illustrated in the case of the binomial. Biometrika.

[24] Brown L.D., Cai T.T., DasGupta A. (2001). Interval estimation for a binomial proportion. Stat. Sci..

[25] Weissman D.E., Meier D.E. (2011). Identifying patients in need of a palliative care assessment in the hospital setting. J. Palliat. Med..

[26] Downar J., Goldman R., Pinto R., Englesakis M., Adhikari N.K. (2017). The “surprise question” and identification of palliative care needs among seriously ill patients. CMAJ.

[27] Courtright K.R., Madden V., Bayes B., Chowdhury M., Whitman C., Small D.S., Harhay M.O., Parra S., Cooney-Zingman E., Ersek M. (2024). Default palliative care consultation for seriously ill hospitalized patients: A pragmatic cluster randomized trial. JAMA.

[28] Earl T., Katapodis N., Schneiderman S. (2020). Care Transitions. Making Healthcare Safer III: A Critical Analysis of Existing and Emerging Patient Safety Practices.

[29] Centers for Disease Control and Prevention (CDC) (2021). Traumatic Brain Injury & Concussion: Discharge Instructions (Adults).

[30] Kwok R., Dinh M., Dinh D., Chu M. (2009). Improving adherence to asthma clinical guidelines and discharge documentation from emergency departments: Implementation of a dynamic and integrated electronic decision support system. Emerg. Med. Australas..

[31] Patterson B.W., Pulia M.S., Ravi S., Hoonakker P.L.T., Schoofs Hundt A., Wiegmann D., Wirkus E.J., Johnson S., Carayon P. (2019). Scope and influence of electronic health record-integrated clinical decision support in the emergency department: A systematic review. Ann. Emerg. Med..

[32] Muhindo M.K., Bress J., Kalanda R., Armas J., Danziger E., Kamya M.R., Butler L.M., Ruel T.D. (2021). Implementation of a newborn clinical decision support software (NoviGuide) in a rural district hospital in Eastern Uganda: Feasibility and acceptability study. JMIR Mhealth Uhealth.

